# 8-*p*-Hdroxybenzoyl Tovarol Induces Paraptosis Like Cell Death and Protective Autophagy in Human Cervical Cancer HeLa Cells

**DOI:** 10.3390/ijms160714979

**Published:** 2015-07-02

**Authors:** Cui Zhang, Yingnan Jiang, Jin Zhang, Jian Huang, Jinhui Wang

**Affiliations:** School of Traditional Chinese Materia Medica, Shenyang Pharmaceutical University, Shenyang 110016, China; E-Mails: zhangcui8534@163.com (C.Z.); yingnanjiang1996@126.com (Y.J.); jinjinlovemama@163.com (J.Z.)

**Keywords:** 8-*p*-hdroxybenzoyl tovarol, germacrane-type sesquiterpenoid, *Ferula dissecta*, HeLa cell, paraptosis, autophagy

## Abstract

8-*p*-Hdroxybenzoyl tovarol (TAW) is a germacrane-type sesquiterpenoid that can be isolated from the roots of *Ferula dissecta* (Ledeb.) Ledeb. In this study, the growth inhibitory effects induced by TAW were screened on some types of tumor cells, and the mechanism was investigated on TAW-induced growth inhibition, including paraptosis and autophagy in human cervical cancer HeLa cells. TAW-induced paraptosis involved extensive cytoplasmic vacuolization in the absence of caspase activation. Additionally, TAW evoked cell paraptotic death mediated by endoplasmic reticulum (ER) stress and unfolded protein response (UPR). Autophagy induced by TAW was found to antagonize paraptosis in HeLa cells. This effect was enhanced by rapamycin and suppressed by the autophagy inhibitor, 3-methyladenine (3MA). Loss of beclin 1 (an autophagic regulator) function led to promote ER stress. Taken together, these results suggest that TAW induces paraptosis like cell death and protective autophagy in HeLa cells, which would provide a new clue for exploiting TAW as a promising agent for the treatment of cervical cancer.

## 1. Introduction

Natural products have been the main source of traditional medicine since ancient time. Today, numerous compounds from natural plants are reported to possess growth inhibitory effects on tumor cells. The *Ferula* genus, belonging to the family of Apiaceae, has been reported to be rich in biologically active compounds such as terpenoid coumarins and sesquiterpene derivatives [1,2,3,4]. Particularly, these compounds have been proven to be cytotoxic on several cancer cell lines and seem to be promising natural products for treatment of human cancers [5,6]. 8-*p*-Hdroxybenzoyl tovarol (TAW), a germacrane-type sesquiterpenoid, was isolated from the roots of *Ferula dissecta* (Ledeb.) Ledeb. However, no further investigations have been carried out on its effects on tumor cells, and the mechanisms underlying the growth inhibitory effects of TAW are still unclear so far.

Cervical cancer is the most common malignancy of the female reproductive system. Although neoadjuvant chemotherapy, along with concurrent chemo- and radiotherapies have benefited the majority of patients, survival in women with recurrent or metastatic cervical cancer remains poor. Resistance of cancer to chemotherapy is one of the primary causes of treatment failure [7,8]. Thus, novel anticancer drugs to combat cervical cancer are needed.

Up to now, cell death could be classified into apoptosis, autophagy, necrosis, cornification and tentative definitions of atypical cell death modalities such as paraptosis, mitotic catastrophe, anoikis, excitotoxicity, wallerian degeneration, pyroptosis, pyronecrosis, entosis *etc.* [9]. Among these types of cell death, at least three of them, *i.e.*, apoptosis, autophagy and paraptosis, belong to programmed cell death (PCD) based on their morphological features [10]. The PCD observed during development and tissue homeostasis has been classified morphologically into three main types [11]: type 1, also known as nuclear or apoptotic; type 2 or autophagic; and type 3, a non-lysosomal vacuolated degeneration, also being called paraptosis [12]. Apoptosis is a caspase-dependent process and exhibits morphological features including nuclear fragmentation, cell shrinkage, chromatin condensation, membrane blebbing, and the formation of apoptotic bodies [13]. Autophagy, specific to eukaryotic cells, is a dynamic process involving the sequestration of plasmatic portions and intracellular organelles into double-membrane vacuoles called autophagosomes. Autophagy has many crucial physiological functions and can trigger lysosome-dependent protein degradation, organelle turnover, and removal [14]. Paraptosis is a kind of PCD that has not been well characterized. It was originally introduced in 2000 to describe a form of PCD morphologically and biochemically different from apoptosis [12]. The main feature of paraptosis consists of cytoplasmic vacuolization that begins with progressive swelling of mitochondria and/or endoplasmic reticulum (ER). Paraptosis typically does not respond to caspase inhibitors nor does it involve activation of caspases, formation of apoptotic bodies, or other characteristics of apoptotic morphology. In contrast to apoptosis, Bcl-X_L_ is also not involved in paraptosis.

In the present study, we demonstrated for the first time that TAW induces paraptosis characterized by cell death in conjunction with cytoplasmic vacuolation, no involvement of caspase activation and DNA fragmentation, dilated ER and mitochondria, and induction of ER stress and unfolded protein response (UPR) signaling markers. In addition, our results showed that TAW also induced autophagy in HeLa cells, and autophagy antagonized paraptosis. Thus, these results suggest that TAW induces paraptosis like cell death and protective autophagy in HeLa cells.

## 2. Results

### 2.1. 8-p-Hdroxybenzoyl Tovarol (TAW) Induces Cytoplasmic Vacuolization and Cell Death in HeLa Cells

**Figure 1:**
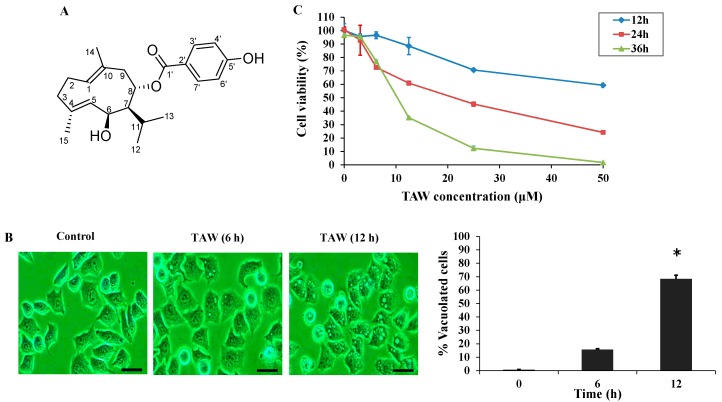
8-*p*-Hdroxybenzoyl tovarol (TAW) induces cytoplasmic vacuole formation in HeLa cells. (**A**) The chemical structures of TAW; (**B**) Light micrograph images (×200 magnification) of these cancer cells with or without TAW treatment. Cells were treated with 18 μM TAW (6, 12 h) for HeLa cells. Bar = 20 μm. And the percentage of vacuolated cells was measured using light microscope. Data are expressed as mean ± S.E.M. * *p* < 0.05 *vs.* control group; (**C**) Results of MTT (3-(4,5-dimethyl-2-thiazolyl)-2,5-diphenyl-2-H-tetrazolium bromide) assay of cell viability. Cells were incubated with escalating concentrations (0–50 μM) of TAW for 12, 24 and 36 h. The data are presented as the mean ± S.E.M. of the results from three independent experiments.

In HeLa cells, extensive cellular vacuolization could be detected under the light microscope after TAW treatment (Figure 1B). The development of cytoplasmic vacuolization in HeLa cells could be described as follows: in TAW-treated cells, small vacuoles appeared (6 h treatment) and then gradually fused into giant vacuoles (12 h treatment). After 24 h treatment, some of the cells shrunk and became detached from the culture plate with intracellular vacuole surrounding the cell nucleus. The effects of TAW on cell viability of HeLa cells were shown in Figure 1C. TAW inhibited HeLa cell growth in a time- and dose-dependent manner, and the IC_50_ value of TAW was 18.8 μM in HeLa cells for 24 h, compared with that of 14.8 μM 5-fluorouracil.

### 2.2. 8-p-Hdroxybenzoyl Tovarol (TAW) Induces Paraptosis Like Cell Death in HeLa Cells

**Figure 2:**
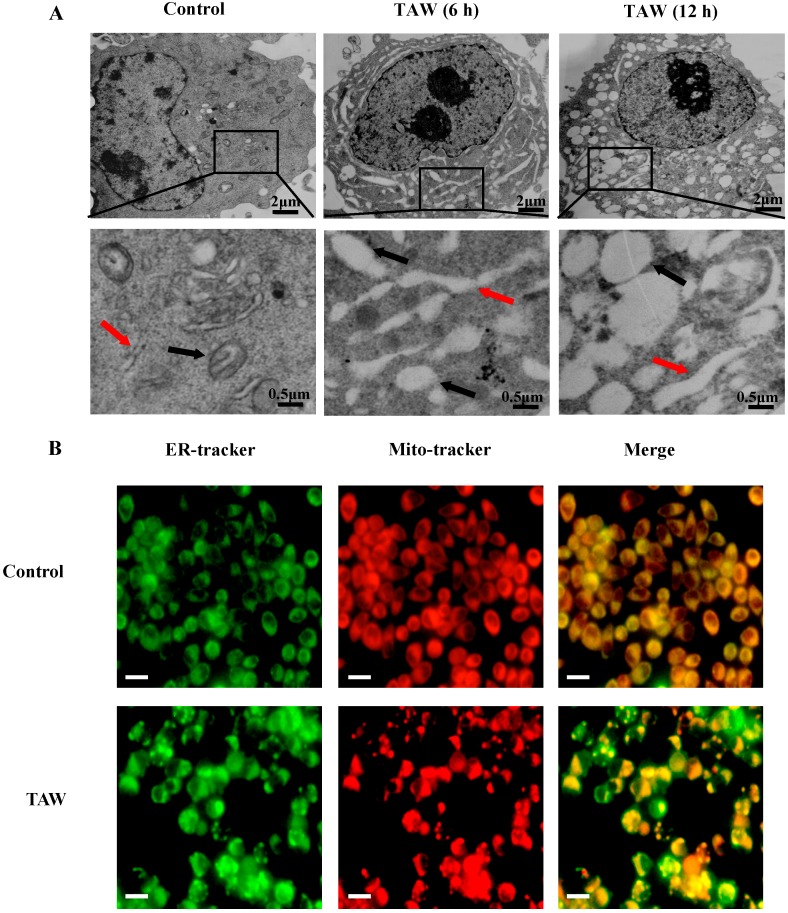

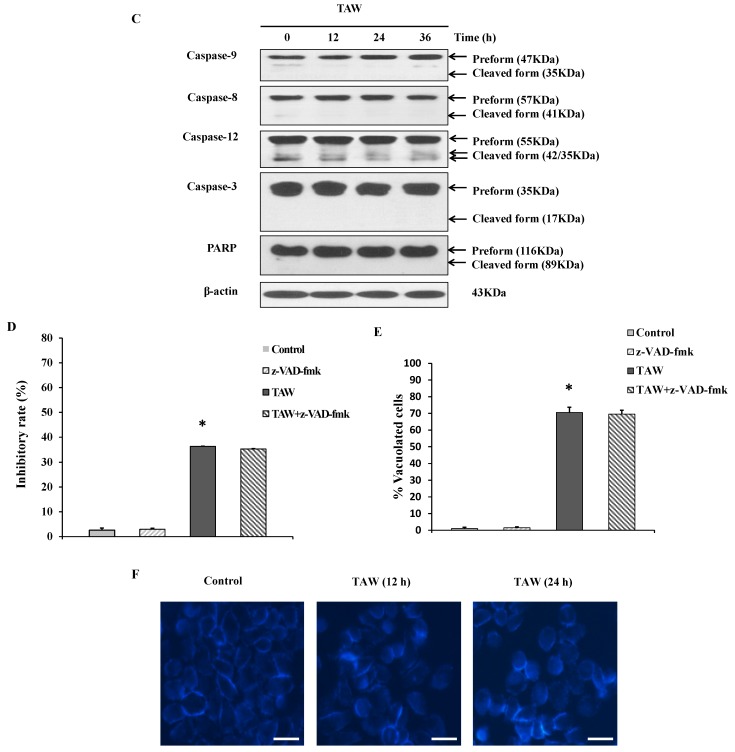
8-*p*-Hdroxybenzoyl tovarol (TAW) induces paraptosis like cell death in HeLa cells. (**A**) Electron microscopy showing ultrastructure of HeLa cells untreated and treated with 18 μM TAW for 6 and 12 h. Control cells with normal mitochondrial and ER. Cells after 6 and 12 h treatment, showing both mitochondrial and ER swelling. Red arrows represent ER, black arrows represent mitochondria; (**B**) Cells incubated with vehicle (control) or 18 μM TAW for 12 h then stained with ER-tracker and Mito-tracker, and observed by fluorescence microscope (×200 magnification). Bar = 20 μm; (**C**) HeLa cells were lysed after treatment with 18 μM TAW for 12, 24, and 36 h, and the protein levels of caspase-3, 8, 9, 12 and PARP were detected by western blot analysis. β-Actin was used as an equal loading control; (**D**,**E**) Cells were pretreated with z-VAD-fmk at 20 μM for 1 h before treatment of TAW at 18 μM for 12 h, then the inhibitory rate was measured using an MTT assay (**D**), and the percentages of vacuolated cells were measured (**E**). Data are expressed as mean ± S.E.M. * *p* < 0.05 *vs.* control; (**F**) The cells were treated with TAW (18 μM) for 12 and 24 h then stained with Hoechst 33258 and observed by fluorescence microscopy (×200 magnification). Bar = 20 μm.

The TAW-induced cytoplasmic vacuolization was further observed by the transmission electron microscope (Figure 2A). The vacuoles appeared clear in HeLa cells treated with 18 μM TAW for 6 h, and no cytoplasmic material was observed in the vacuoles. At 12 h of TAW treatment, fusion among the swollen mitochondria and ER were further progressed. To further characterize the morphological dynamics of the cytoplasmic vacuolization process, experiments were performed in HeLa cells by using Mito-tracker and ER-tracker stains. As shown in Figure 2B, vacuoles could be observed through mitochondria and ER staining in HeLa cells treated with TAW. Cytoplasmic vacuolization and enlarged mitochondria and/or ER have been reported to be the typical features of paraptosis [12]. Paraptosis typically does not respond to caspase inhibitors nor does it involve activation of caspases, the formation of apoptotic bodies, or DNA fragmentation [11]. Next, to examine the involvement of caspase activation, cells were treated with TAW, then caspase-3, 8, 9, 12 and downstream poly-ADP-ribose polymerase (PARP) protein levels were measured. As a result of treatment, intact caspase-3, 8, 9, 12 and PARP protein levels were not changed, and cleaved caspase-3, 8, 9, 12 and cleaved PARP proteins were not detected (Figure 2C). When cells were pretreated with the broad spectrum pan-caspase inhibitor z-VAD-fmk before treatment of TAW, the percentages of dead cells (Figure 2D) and vacuolated cells (Figure 2E) were not altered, regardless of pretreatment with z-VAD-fmk. Moreover, Hoechst 33258 staining assay (Figure 2F) showed that no obvious morphological alterations were caused in the nucleus of TAW-treated HeLa cells at different time points. Taken together, these results demonstrate that TAW induces paraptosis like cell death in HeLa cells.

### 2.3. 8-p-Hdroxybenzoyl Tovarol (TAW) Treatment Induces Depletion of Mitochondrial Membrane Potential (MMP)

**Figure 3:**
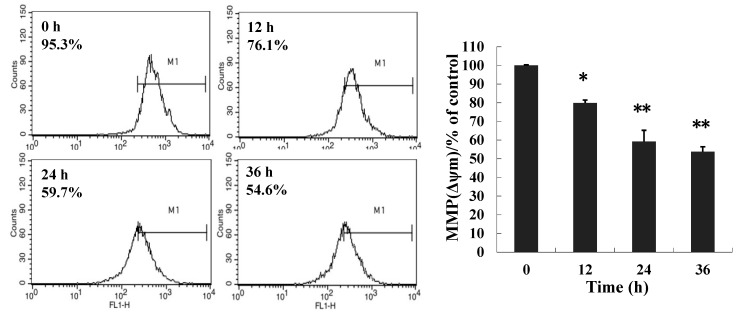
Loss of mitochondrial membrane potential induced by 8-*p*-hdroxybenzoyl tovarol (TAW). HeLa cells untreated or treated with TAW (18 μM) for 12, 24 and 36 h were stained with Rhodamine 123 dye and change in fluorescent intensity was assessed by flow cytometry. Data represent the mean ± S.E.M. of three replicate independent experiments. * *p* < 0.05 *vs.* control group, ** *p* < 0.01 *vs.* control group.

To examine the effects of TAW on mitochondrial membrane potential, HeLa cells untreated or treated with TAW for 12, 24 and 36 h were stained with Rhodamine 123 dye and change of fluorescent intensity was assessed by the flow cytometry. It suggests that TAW treatment significantly reduces the MMP of HeLa cells (Figure 3).

### 2.4. 8-p-Hdroxybenzoyl Tovarol (TAW) Induced Vacuolation Is Reversed by Treatment with Cycloheximide in HeLa Cells

**Figure 4:**
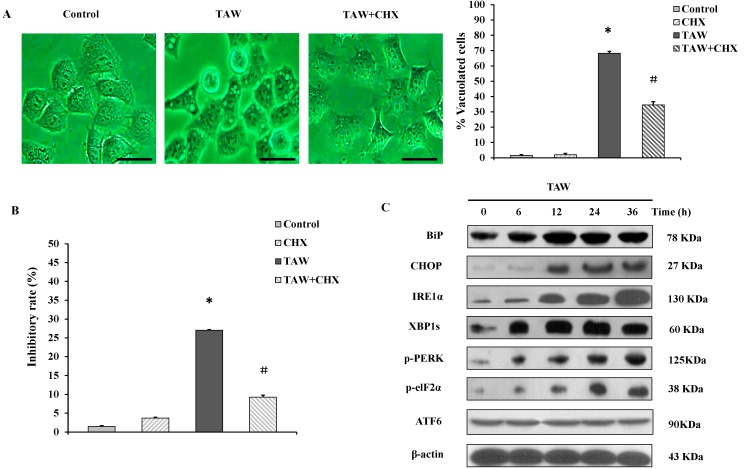
The involvement of prevention of vacuolation by cycloheximide, ER-stress and UPR signaling pathway in 8-*p*-hdroxybenzoyl tovarol (TAW) induced paraptosis in HeLa cells. (**A**,**B**) Cells were pretreated with CHX (1.25 μM, 1 h), then treated with TAW at 18 μM for 12 h. The light micrograph images (×400 magnification, Bar = 20 μm) and the percentages of vacuolated cells were measured using a light microscope (**A**), and the inhibitory rate was measured using an MTT assay (**B**). Data are expressed as mean ± S.E.M. * *p* < 0.05 *vs.* control group. ^#^ *p* < 0.05 *vs.* Cells treated with TAW alone; (**C**) HeLa cells were treated with TAW (18 μM) for indicated time points prior to preparation of lysates, followed by immunoblotting to detect the protein levels of BiP, CHOP, IRE1α, XBP1s, p-PERK, p-eIF2α, and ATF6. β-Actin was used as an equal loading control.

As shown in Figure 4A, halting of protein synthesis by addition of translation inhibitor cycloheximide (CHX) at 1.25 μM could significantly inhibit the formation of cytoplasmic vacuolization induced by TAW and decreased the number of cells with cytoplasmic vacuolization, suggesting that cytoplasmic vacuolization was interrupted by translation inhibitor, another characteristic of paraptosis [12]. Moreover, the pretreatment of HeLa cells with 1.25 μM CHX effectively decreased the level of TAW-induced cell death (Figure 4B). Taken together, these results demonstrate that TAW induces paraptosis like cell death in HeLa cells, and an active role of ER protein loads in vacuolation process in TAW-induced paraptosis.

### 2.5. 8-p-Hdroxybenzoyl Tovarol (TAW)-Mediated ER-Stress and Unfolded Protein Response (UPR) Lead to Paraptosis Like Cell Death

In view of dilation of the ER-lumen and the percentages of dead cells and vacuolated cells rescued by CHX involved in TAW-induced paraptosis, we reasoned that TAW induced vacuolization may be preceded by induction of ER-stress. We performed a time course experiment to study ER-stress and activation of UPR by evaluating expression of certain ER resident proteins involved in protein folding. Immunoblot analysis of the cells demonstrated that TAW upregulated signature ER-stress markers glucose-regulated protein BiP/GRP78 and C/EBP-homologous protein CHOP (Figure 4C). To further validate the capacity of TAW to induce ER-stress, we asked whether ER stress-induced UPR signaling pathways are evoked upon TAW stimulation. It is clear to note that TAW treatment caused increased expression of endoribonuclease, inositol requiring enzyme 1 (IRE1α), and the splicing of X-box binding protein (XBP1s), indicating the activation of the IRE1 branch of UPR signaling. Additionally, an increase in expression of auto-phosphorylated PKR-like endoplasmic reticulum (ER) resident kinase (p-PERK) and PERK-mediated phosphorylation of eukaryotic initiation factor (p-eIF2α) were also observed, suggesting that TAW engages the PERK branch of UPR signaling. However, there was no change in ATF6 level, indicating that the ATF6 branch of UPR signaling is not involved in (Figure 4C). Altogether, these results indicate that TAW-mediated ER-stress and UPR signaling leads to paraptosis.

### 2.6. 8-p-Hdroxybenzoyl Tovarol (TAW) Induces Autophagy in HeLa Cells

**Figure 5:**
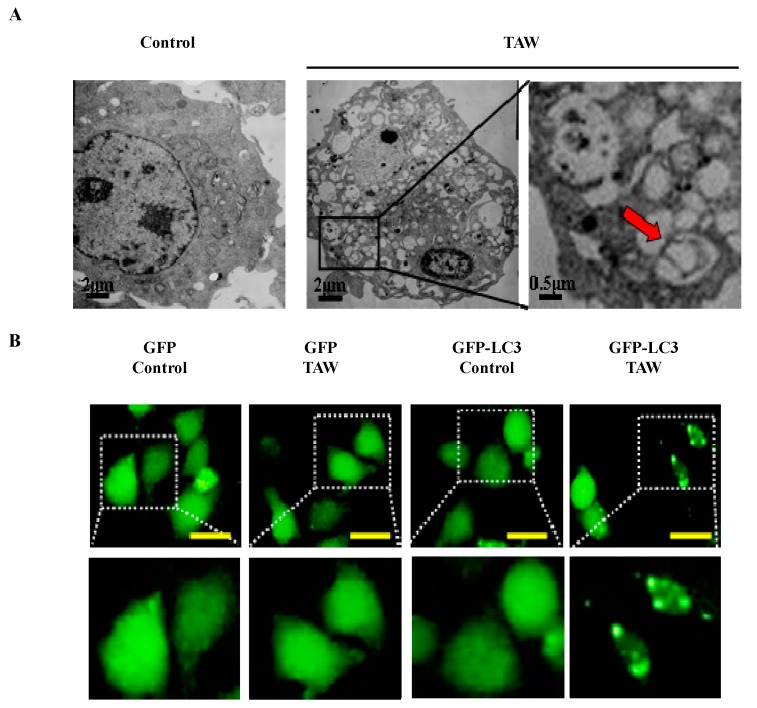

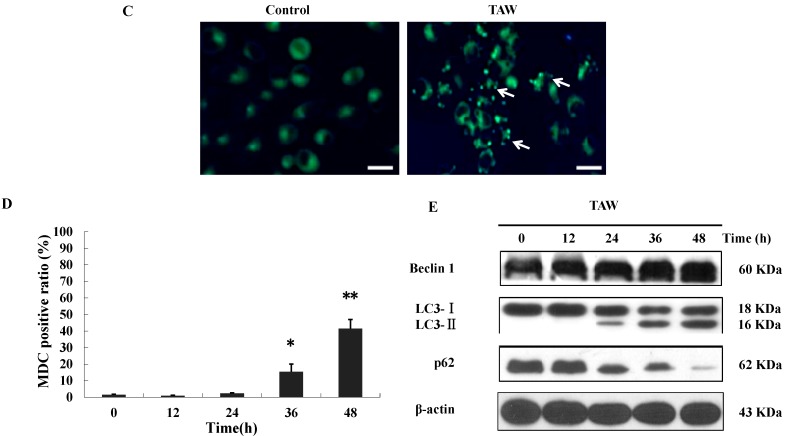
8-*p*-hdroxybenzoyl tovarol (TAW) induces autophagy in HeLa cells. (**A**) HeLa cells were untreated or treated with 18 μM TAW for 36 h, and formation of autophagic vacuoles was examined by TEM analysis. Red arrows represent double-membrane autophagosome. The data was representative of three independent experiments; (**B**) HeLa cells were transfected with a pEGFP-LC3 plasmid. After 24 h, cells were untreated or indicated 18 μM of TAW for another 36 h. Formation of EGFP-LC3 puncta was visualized by a fluorescence microscope (×400 magnification, Bar = 20 μm). The data was representative of three independent experiments; (**C**) Cells were cultured with 18 μM TAW for 36 h and then observed by fluorescence microscopy with MDC staining (×200 magnification, Bar = 20 μm). Arrows indicate cells containing autophagolysosomes; (**D**) The cells were treated with 18 μM TAW for 12, 24, 36 and 48 h, and quantitative analysis detected a positive ratio of MDC staining by flow cytometric analysis, *n* = 3, means ± S.E.M. * *p* < 0.05 *vs.* control group, ** *p* < 0.01 *vs.* control group; (**E**) HeLa cells were lysed after treatment with 18 μM TAW for 12, 24, 36 and 48 h, and the protein levels of beclin 1, LC3 and p62 were detected by western blot analysis. β-Actin was used as an equal loading control.

To determine the effect of TAW on autophagy in HeLa cells, firstly the ultrastructure of cells was examined by transmission electron microscopy. The HeLa cells treated with TAW (18 μM) for 36 h, and ultrastructural analysis revealed an increased number of autophagosomes in TAW-treated HeLa cells (Figure 5A). As shown in Figure 5B, HeLa cells were transfected with a pEGFP-LC3 plasmid, and exogenous EGFP-LC3 puncta were also increased in TAW-treated HeLa cells. The cells were observed with monodansylcadaverine (MDC) staining by fluorescence microscope. Compared with the control group, TAW treatment caused a marked increase in the number of MDC labeled autophagolysosomes in the cells (Figure 5C). Flow cytometric analysis also showed that the MDC-positive cells caused by treatment with TAW were increased in a time-dependent manner (Figure 5D). Western blot analysis revealed that the upregulation of beclin 1, the conversion from LC3 I to LC3 II and the downregulation of p62 were detected in TAW treated cells in a time-dependent manner (Figure 5E). These results demonstrate that TAW induces autophagy in HeLa cells.

### 2.7. Autophagy Antagonizes Paraptosis in 8-p-Hdroxybenzoyl Tovarol (TAW) Treated HeLa Cells

**Figure 6:**
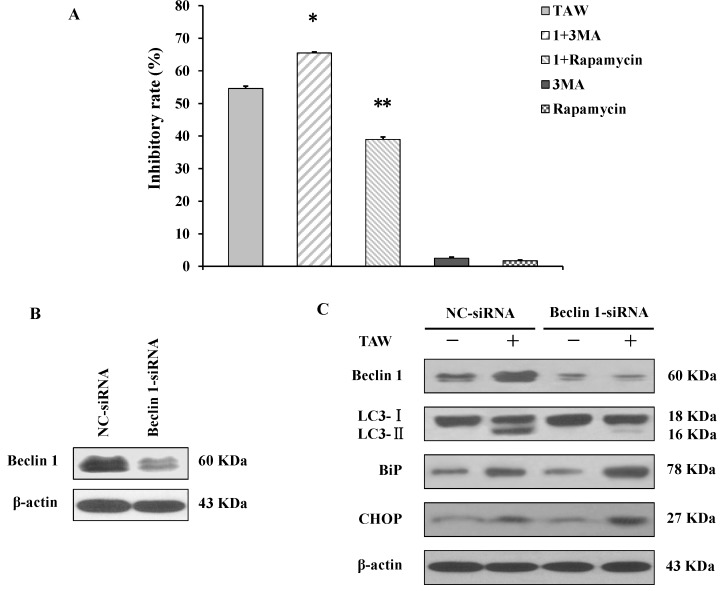
8-*p*-Hdroxybenzoyl tovarol (TAW)-induced autophagy antagonizes paraptosis in HeLa cells. (**A**) Cells were incubated with 18 μM TAW in the presence or absence of 3MA (1 mM) or rapamycin (1 nM) for 24 h, and the inhibitory rate was measured using an MTT assay. Data are expressed as mean ± S.E.M. * *p* < 0.05 *vs.* control group, ** *p* < 0.01 *vs.* control group; (**B**) HeLa cells were transfected with either non-specific siRNA (NC-siRNA) or beclin 1-siRNA, and beclin 1 protein was detected 24 h after transfection by western blot analysis. β-Actin was used as an equal loading control; (**C**) The transfected HeLa cells were lysed after treatment with 18 μM TAW for 24 h; then the expression of beclin 1, conversion from LC3 I to LC3 II, BiP and CHOP were detected by western blot analysis. β-Actin was used as an equal loading control.

TAW is therefore able to induce paraptosis and autophagy in HeLa cells. To find out the relationship between paraptosis and autophagy, the specific autophagic inhibitor 3-methyladenine (3MA) and the autophagic agonist rapamycin were applied. The MTT assay showed that TAW inhibited cell growth significantly, but this inhibition was partially augmented by 3MA and was reversed by rapamycin (Figure 6A). Moreover to elucidate the role of autophagy in TAW-induced paraptosis, the expression of the autophagy-associated marker protein beclin 1 in HeLa cells was knocked down by application of short interfering RNA (siRNA). After transfection, the changes at the protein level were evaluated (Figure 6B). TAW-induced up-regulation of beclin 1 was reversed, and the conversion from LC3 I to LC3 II was also suppressed, demonstrating that the autophagy induced by TAW was markedly inhibited (Figure 6C). Next, the loss of beclin 1 further enhanced TAW-induced upregulation of the ER-stress markers such as BiP and CHOP (Figure 6C). These results further demonstrate that autophagy antagonizes paraptosis in HeLa cells treated with TAW.

## 3. Discussion

Natural products isolated from plants or medicinal herbs have been invaluable resources of antineoplastic agents or lead compounds for new drug development [15]. There are at least 250,000 species of plants out of which more than one thousand plants have been found to possess significant anticancer properties [16]. *Ferula dissecta*, has a long history of application in the treatment of digestive diseases and arthritis in Uygur medicine. Syreiteate A and syreiteate B, two constituents of *Ferula dissecta*, have been reported to have growth inhibitory activity against cervical cancer HeLa cell line [17]. 8-*p*-Hdroxybenzoyl tovarol (TAW) is an active germacrane-type sesquiterpenoid which was isolated from the roots of *Ferula dissecta*. In this study, the cytotoxic effect and the underlying mechanism of TAW are examined. To our best knowledge, this is the first report as to TAW-induced cytotoxicity and the underlying mechanism on human cervical cancer HeLa cells.

Classical apoptosis is caspase-dependent programmed cell death (CD-PCD). Accumulating evidence in the literature supports that several types of caspase-independent programmed cell death (CI-PCD) exist in the cells, such as autophagy, cornification, mitotic catastrophe, anoikis, paraptosis, excitotoxicity, Wallerian degeneration and programmed necrosis [18]. Caspase-mediated apoptosis is a major hindrance to tumour growth and metastasis. Moreover, many tumour cells can unexpectedly survive the activation of caspases. As a result, caspase-independent cell death programs are gaining increasing interest among cancer researchers [19]. Therefore, CI-PCD may play an important role in inducing cell death and be the major death style in many tumor cells. Paraptosis, a new type of CI-PCD, is characterized mainly by a process of cytoplasmic vacuolization that begins with the physical enlargement of mitochondria and/or endoplasmic reticulum. This PCD does not involve the apoptotic characteristics of pyknosis, DNA fragmentation or caspase activation, and is known to require new protein synthesis [20]. Studying paraptosis will not only be helpful to clarify the mechanism of cell death but also to improve therapies for cancer treatment. Therapies based on the induction of non-apoptotic cell deaths such as paraptosis might be possible approaches to suppress the multi-drug resistant phenotype often associated with resistance to apoptosis [21]. In our study TAW induced cytoplasmic vacuolation, which was derived from the swelling mitochondria and ER in HeLa cells, with no characteristics of apoptosis, such as DNA fragmentation, apoptotic bodies and activation of caspases. These suggest that this kind of cell death induced by TAW is different from apoptosis. These results indicate that TAW induces paraptosis like cell death in human cervical cancer HeLa cells.

Reduction in mitochondrial membrane potential (MMP) can be a checkpoint of apoptosis as it can cause the release of apoptogenic molecules from the intermembrane space into the cytoplasm [22]. In the present study we have observed significant reduction in MMP after exposure to TAW. But lack of caspase activation also was observed suggesting that loss of MMP is not apoptosis in TAW induced cell death. Depletion of MMP was also observed without AIF (apoptosis inducing factor) release in calcium induced paraptotic cell death in Jurkat cells [23]. Summing up these results also suggest that TAW induces paraptosis like cell death in HeLa cells.

The endoplasmic reticulum provides a specialized niche for protein maturation that includes posttranslational modification, proper folding, attainment of native state and finally their transport [24]. ER stress occurs when the rate of translation of proteins exceeds their proper rate of folding [25]. This stressful situation induces a transcriptional program called the unfolded protein response (UPR). The UPR causes attenuation of global translation and selectively upregulates chaperones that act to clear unfolded proteins from the stressed ER [26]. The UPR is fundamentally cyto-protective, but the compensatory mechanisms may not be able to fully sustain ER function and the decompensation will activate the cell death pathway if the stress is too severe or lasts for too long [27]. During the UPR, signaling branches are activated by three parallel sensors of stress in the ER membrane: (1) protein kinase RNA-like endoplasmic reticulum kinase (PERK); (2) activation transcription factor 6 (ATF6) and (3) inositol requiring enzyme 1 (IRE1) [28]. The kinase PERK directly inhibits mRNA translation by phosphorylating translation initiation factor 2α (eIF2α) [29]. ATF6 transduces ER stress after its cleavage, which yields a fragment that translocates to the nucleus to regulate gene expression [30]. Active IRE1 lead to an unconventional splicing of the transcription factor XBP1 triggering the removal of 26 nucleotides and the translation of an active transcription factor [31]. ER dilatation and vacuolization are processes that define type III or paraptotic-like cell death and, recently, a relationship has been observed between this type of cell death and the ER stress induced by proteasome inhibitors [32]. Consistently, we show here that TAW-induced paraptosis cell death in HeLa cells, hallmarked by ER-derived vacuolization. Then the TAW induced ER dilatation enhanced the levels of ER-resident chaperone BiP and other markers of ER stress such as CHOP. ER stress trigger UPR signaling pathways via activation of IRE1 and the persistent XBP1 cleavage constitutes the transcription dependent pathway, and global translation inhibition by the elevated eIF2α phosphorylation levels. According to these results, TAW could induce ER stress and UPR in HeLa cells. We pretreated HeLa cells with CHX to suppress ER stress and the percentages of dead cells and vacuolated cells were rescued. These findings suggest that TAW induces paraptotic death in HeLa cells through ER stress and UPR. Moreover, the TAW inhibits caspase-3, caspase-9, caspase-8 and caspase-12 proteins that involve three signaling pathways that trigger apoptosis [27] and triggers a caspase-independent death program. Therefore, the paraptotic-like cell death may be a backup cell death pathway that takes place when a critical amount of unfolded proteins is reached and, concomitantly, the apoptotic machinery is hindered. This hypothesis is consistent with the information presented in other reports [32].

Autophagy is a cellular digestive process conserved from yeast to mammals. Depending on the cellular context, autophagy may promote cell survival by preventing the accumulation of deleterious products and organelles [33], or serve as a type of programmed cell death through cell lysis [34]. The self-digestion not only provides cells with nutrients during fasting, but also, it can rid the cell of superfluous or damaged organelles, misfolded proteins and invading microorganisms. Autophagy therefore is important for stress resistance [35]. Dysfunction of autophagy results in accumulation of damages and finally leads to cell death [35]. In addition, autophagy can also be induced in a response to chemotherapy and has been considered as an important mechanism contributing to drug resistance, and the autophagic process may protect cancer cells from toxicity. Therefore, the inhibition of autophagy may result in a greater susceptibility to cell death-inducing mechanisms [36]. In our study, we detected the morphological changes and the expression of autophagy maker genes Beclin 1, LC3 II, and p62 in HeLa cells treatment with TAW. Our detailed results show that TAW induces autophagy in HeLa cells. Then to explore the relationship between paraptosis and autophagy in TAW-induced HeLa cells, the specific autophagic inhibitor 3MA and the autophagic agonist rapamycin were applied. An MTT assay showed that the cytotoxic effect of TAW-treatment HeLa cells was potentiated by an inhibition of autophagy. Furthermore, after transfection Beclin 1-siRNA, the results demonstrate that a blockade of autophagy can increase the expression of the ER stress marker genes BiP and CHOP. These results suggest that ER stress-mediated paraptosis cell death after TAW treatment is potentiated by an inhibition of autophagy.

In summary, this study describes HeLa cells growth inhibitory activity of a germacrane-type sesquiterpenoid 8-*p*-hdroxybenzoyl tovarol (TAW), isolated from the roots of *Ferula dissecta*. The mechanisms are TAW induced ER stress-mediated paraptosis like cell death and protective autophagy in human cervical cancer HeLa cells. These results would provide a new clue for exploiting TAW as a promising agent for the treatment of cervical cancer.

## 4. Experimental Section

### 4.1. Plant Materials and Reagents

Biological materials: the roots of *F. dissecta* were collected in Tuoli, Xinjiang of China in June 2007 and identified by Yong Tan (Shihezi University of China). A voucher specimen (No. 20071018001) is deposited in School of Pharmacy, Shihezi University. *Ferula dissecta* (Ledeb.) Ledeb are widely distributed along the Junggar Basin in Xinjiang. *Ferula dissecta* (Ledeb.) Ledeb used in this study were picked in the hills, plains and valleys of Xinjiang Toli County randomly (Toli County located in the west of Junggar Basin). All these locations do not require any specific permission, and none of the field studies involved endangered or protected species.

Reagents: 3-(4,5-dimethyl-2-thiazolyl)-2,5-diphenyl-2-H-tetrazolium bromide (MTT) (Sigma, Tokyo, Japan, M5655), cycloheximide (Sigma, A6185), monodansylcadaverine (MDC) (Sigma, 30432), Rhodamine 123 (Sigma, R8004), Rapamycin (Sigma, R8781), 3-Methyladenine (Sigma, M9281) were purchased from Sigma. Endoplasmic reticulum (ER)-tracker (Invitrogen, Carlsbad, CA, USA, E34250), Mitochondrion (Mito)-tracker (Invitrogen, M7514), Lipofectamine 2000 (Invitrogen, 11668), Lipofectamine RNAiMAX (Invitrogen, 13778) were purchased from Invitrogen. Hankʼs Balanced Salt Solution with calcium and magnesium (HBSS/Ca/Mg, Gibco, Grand Island, NY, USA, #14025-092) was purchased from Gibco.

Antibodies used in this study were against: BiP (Cell Signaling, Boston, MA, USA, #3177), CHOP (Cell Signaling, #2895), IRE1α (Cell Signaling, #3294), XBP1s (Cell Signaling, #12782), phospho-PERK (Santa Cruz, Buda, TX, USA, sc-32577), phospho-elF2α (Cell Signaling, #3398), ATF6 (Abcam, Cambridge, UK, ab37149), caspase-3 (Cell Signaling, #9665), caspase-9 (Cell Signaling, #9508), caspase-8 (Cell Signaling, #9746), caspase-12 (Cell Signaling, #2202), PARP (Cell Signaling, #9532), Beclin 1 (Cell Signaling, #3495), LC3 I/II (Cell Signaling, #4108), p62 (Cell Signaling, #8025), β-Actin (Proteintech, Chicago, IL, USA, 66009-1-lg), anti-rabbit lgG (Cell Signaling, #7074), anti-mouse lgG (Cell Signaling, #7076).

### 4.2. Extraction and Isolation of TAW

The air-dried roots (1.45 kg) of *F. dissecta* were refluxed with 95% Ethanol (15 L) three times for 2 h. The extract was concentrated under reduced pressure to yield an Ethanol extract (240 g). A part of the Ethanol extract (240 g) was subjected to chromatography over silica gel, eluted with petroleum ether (PE) with an increasing amount of EtOAc to afford fraction E (PE-EtOAc, 100:6, 1.2 g). Fr. E was then separated by silica gel (PE-EtOAc, 100:7.5) to give the crystal block of TAW (30.0 mg). Its chemical structure (Figure 1A) was identified through a comparison of its spectral data (MS, IR, ^1^H- and ^13^C-NMR, single crystal X-ray) with those published previously [37], and its purity was above 98% as determined by HPLC-UV.

### 4.3. Cell Culture

HeLa cells were obtained from American Type Culture Collection (ATCC, Manassas, VA, USA) and were cultured in PRMI-1640 (Gibco, Life Technologies, Carlsbad, CA, USA) with 10% fetal bovine serum (FBS) (Biolianshuo, Yuanheng, China) at 37 °C in 5% CO_2_.

### 4.4. Cell Viability Assay

The inhibitory effect of TAW on HeLa cells viability was measured by MTT assay. The cells were dispensed in 96-well flat-bottom microtiter plates (BD Falcon, Austin, TX, USA) at a density of 4.0 × 10^4^ cells per well. After 24 h incubation, they were treated with various concentrations of TAW for indicated time points. After that, the cells were incubated with 5.0 mg/mL MTT solution at 37 °C for 4 h. The resulting crystal was dissolved in 100 μL DMSO and the optical density was measured by using a micro-plate reader (Thermo Multiskan MK3, Thermo scientific, Helsinki, Finland). If required, the pan-caspase inhibitor z-VAD-fmk (20 μM), cycloheximide (CHX, 1.25 μM), 3-Methyladenine (3MA, 1 mM) or rapamycin (1 nM) were added to HeLa cells 1 h before TAW treatment.

### 4.5. Endoplasmic Reticulum (ER)-Tracker and Mito-Tracker

Endoplasmic reticulum or mitochondrion staining was performed according to the instructions of the manufacturer of ER-tracker kit or Mito-tracker kit with slight alteration. Briefly, cells were washed with HBSS or PBS and then incubated in pre-warmed ER-tracker (1 μM) or Mito-tracker (20 nM) dye solution for approximately 30 min at 37 °C. After HBSS or PBS washes, cells were then observed using a fluorescence microscopy (Olympus, Tokyo, Japan).

### 4.6. Hoechst 33258, MDC and Rhodamine 123 Staining

After incubation with TAW for indicated time points, the cells were stained with Hoechst 33258, MDC or Rhodamin 123 at 37 °C for 30 min, and then the morphology was observed by a fluorescence microscopy (Olympus, Tokyo, Japan). Mitochondrial membrane potential was measured by Rhodamine 123 dye, and the samples were subsequently analysed by a FACSCalibur flow cytometry (Becton Dickinson, Franklin Lakes, NJ, USA). The MDC-positive cells were also analysed by a FACSCalibur flow cytometry.

### 4.7. Transmission Electron Microscopy

The ultra-structure of cytoplasmic vacuolization was observed using a Philips Tecnai-12 Biotwin Transmission Electron Microscope similar to previous reports [38]. Briefly, collected cells were fixed with 2% glutaraldehyde for 2 h, washed with PBS and then post-fixed with 1% OsO_4_ for 1.5 h at 4 °C. The samples were then washed and dehydrated with graded alcohol. After dehydration, the samples were infiltrated and embedded in 618 epoxy resin. Ultrathin sections were cut, stained with uranyl acetate and lead citrate and then examined under the transmission electron microscope.

### 4.8. Vacuolated Cell Counting

Vacuolated cells were counted using a method similar to the previous study [39]. Light micrographs were obtained from different fields and the number of vacuolated cells having the total vacuoles occupying area more than 50% of cytoplasmic area was counted for at least 300 cells for each condition.

### 4.9. Western Blot Analysis

The HeLa cells were harvested, washed twice with cold PBS and then lysed in whole cell lysis buffer, supplemented with the proteinase inhibitors 100 µg/mL at 4 °C for 1 h. After 12,000× *g* centrifugation at 4 °C for 10 min, the protein concentration was determined by a BCA Protein Assay Kit (CWBIO, Beijing, China). Equal amounts of total proteins were separated by 12% SDS-PAGE, and transferred onto Immobilon-P Transfer Membrane (Millipore Corporation, Billerica, MA, USA). The membranes were blocked with 5% skimmed milk at room temperature for 1 h, incubated with indicated primary antibodies at 4 °C overnight and horseradish peroxidase (HRP)-conjugated secondary antibody at room temperature for 2 h, then visualized by using ECL reagents.

### 4.10. Transfection of siRNA

Beclin 1 and negative control (NC) siRNAs were purchased from Santa Cruz Biotechnology (Santa Cruz, CA, USA). Cells were transfected with 50 nM Beclin 1-siRNA, or NC-siRNA using Lipofectamine RNAiMAX reagent (Invitrogen) according to the manufacturer’s instructions. The transfected cells were used for subsequent experiments 24 h later.

### 4.11. Transfection of EGFP-LC3 Plasmid

HeLa cells grown to 80% confluency were transfected with an EGFP-LC3 plasmid. Twenty-four hours post transfection, cells were treated with TAW (18 μM) for another 36 h and analyzed by fluorescence microscopy. The EGFP-LC3 plasmid was kindly provided by Bo Liu (Sichuan University, Chengdu, China).

### 4.12. Statistical Analysis

Each experiment was repeated for at least three times and results of three independent experiments were used for statistical analysis. The results were shown as mean ± S.E.M. Statistical comparisons were conducted using Student’s *t*-test in SPSS17.0. *p* < 0.05 was considered as statistically significant.
